# Altered gut microbiota in female mice with persistent low body weights following removal of post-weaning chronic dietary restriction

**DOI:** 10.1186/s13073-016-0357-1

**Published:** 2016-10-03

**Authors:** Jun Chen, Yoshitaka Toyomasu, Yujiro Hayashi, David R. Linden, Joseph H. Szurszewski, Heidi Nelson, Gianrico Farrugia, Purna C. Kashyap, Nicholas Chia, Tamas Ordog

**Affiliations:** 1 Division of Biomedical Statistics and Bioinformatics, Department of Health Sciences Research, Mayo Clinic, 200 1st St SW, Rochester, MN 55905 USA; 2 Enteric NeuroScience Program, Mayo Clinic, 200 1st St SW, Rochester, MN 55905 USA; 3 Division of Gastroenterology and Hepatology, Mayo Clinic, 200 1st St SW, Rochester, MN 55905 USA; 4 Department of Physiology and Biomedical Engineering, Mayo Clinic, 200 1st St SW, Rochester, MN 55905 USA; 5 Department of Surgery, Mayo Clinic, 200 1st St SW, Rochester, MN 55905 USA; 6 Center for Individualized Medicine, Mayo Clinic, 200 1st St SW, Rochester, MN 55905 USA

**Keywords:** Anorexia nervosa, Dietary restriction, Protein-energy malnutrition, Gut microbiota, Insulin-like growth factor 1 (IGF1), Machine learning, Animal model

## Abstract

**Background:**

Nutritional interventions often fail to prevent growth failure in childhood and adolescent malnutrition and the mechanisms remain unclear. Recent studies revealed altered microbiota in malnourished children and anorexia nervosa. To facilitate mechanistic studies under physiologically relevant conditions, we established a mouse model of growth failure following chronic dietary restriction and examined microbiota in relation to age, diet, body weight, and anabolic treatment.

**Methods:**

Four-week-old female BALB/c mice (n = 12/group) were fed ad libitum (AL) or offered limited food to abolish weight gain (LF). A subset of restricted mice was treated with an insulin-like growth factor 1 (IGF1) analog. Food access was restored in a subset of untreated LF (LF-RF) and IGF1-treated LF mice (TLF-RF) on day 97. Gut microbiota were determined on days 69, 96–99 and 120 by next generation sequencing of the V3–5 region of the 16S rRNA gene. Microbiota–host factor associations were analyzed by distance-based PERMANOVA and quantified by the coefficient of determination R^2^ for age, diet, and normalized body weight change (Δbwt). Microbial taxa on day 120 were compared following fitting with an overdispersed Poisson regression model. The machine learning algorithm Random Forests was used to predict age based on the microbiota.

**Results:**

On day 120, Δbwt in AL, LF, LF-RF, and TLF-RF mice was 52 ± 3, –6 ± 1*, 40 ± 3*, and 46 ± 2 % (*, *P* < 0.05 versus AL). Age and diet, but not Δbwt, were associated with gut microbiota composition. Age explained a larger proportion of the microbiota variability than diet or Δbwt. Random Forests predicted chronological age based on the microbiota and indicated microbiota immaturity in the LF mice before, but not after, refeeding. However, on day 120, the microbiota community structure of LF-RF mice was significantly different from that of both AL and LF mice. IGF1 mitigated the difference from the AL group. Refed groups had a higher abundance of *Bacteroidetes* and *Proteobacteria* and a lower abundance of *Firmicutes* than AL mice.

**Conclusions:**

Persistent growth failure can be induced by 97-day dietary restriction in young female mice and is associated with microbiota changes seen in lean mice and individuals and anorexia nervosa. IGF1 facilitates recovery of body weights and microbiota.

**Electronic supplementary material:**

The online version of this article (doi:10.1186/s13073-016-0357-1) contains supplementary material, which is available to authorized users.

## Background

Growth failure including stunting (low height for age), wasting (low weight for height), and being underweight (low weight for age) is a worldwide problem arising from malnutrition characterized by hunger and inadequate food sources or dietary restrictions or from anorexia combined with energy loss or increased metabolic demand. A variety of diseases affecting children and adolescents, such as anorexia nervosa, endocrine disease, chronic kidney and liver disease, congenital heart disease, cystic fibrosis and muscular dystrophies, infections, cancer, malabsorptions, inflammatory diseases, short bowel syndrome, and drugs, may lead to growth failure [1, 2]. Inadequate growth is an indication of systemic dysfunction occurring during a critical phase of development, which has been linked to permanent physical and cognitive deficiencies [1, 3, 4].

Therapeutic food interventions have reduced mortality in childhood malnutrition [5] and, together with behavioral, family-based, and pharmacological treatments, can also achieve weight gain in patients with anorexia nervosa [2, 6]. However, lack of complete and lasting responses and incomplete restoration of growth remain a major problem in all forms of malnutrition and wasting [1, 4, 6–9].

The mechanisms underlying the lack of durable responses remain unclear. One mechanism may be altered gut microbial communities as food is a major determinant of the proportional representation of gut microbes and the genes present in their community [10]. Conversely, microbes affect host metabolic efficiency by extracting and metabolizing dietary components [11, 12] and, once changed due to limited or otherwise altered diet, could influence responses to nutritional therapy. Recently, children with severe or moderate acute malnutrition were found to harbor immature microbiota, i.e. microbiota characteristic of healthy children of a younger age [7, 13]. Immature microbiota persisted despite a transient improvement in response to therapeutic nutritional interventions, paralleling similarly short-lived and partial improvements in anthropometric parameters [7]. Age-discriminatory taxa were found to alter the growth of gnotobiotic mice demonstrating a causal role of microbiota immaturity in undernutrition [13]. Furthermore, microbiota alterations characteristic of lean mice and individuals have recently been reported in patients with anorexia nervosa [9, 14–16]. Thus, intestinal microbiota altered by diet and other disease-related conditions may limit nutritional rehabilitation in patients with malnutrition and wasting of various etiologies.

To facilitate future investigation into the likely complex interactions between quantitative changes in food intake, microbiota, and host physiology [17, 18] in the context of growth failure, suitable animal models are needed. Gnotobiotic mice as recipients of human microbiota transplants have been useful for identifying the function of age-associated and growth-associated taxa [13]. However, they differ from conventionally raised mice and do not necessarily reproduce host physiology [18]. Therefore, in this study we aimed to establish an animal model of persistent low body weight following chronic dietary restriction and investigated microbiota composition in relation to age, food intake, and body weight changes. We studied young post-weaning, rapidly growing female mice as an approximation of anorexia nervosa, which affects mainly girls with a peak onset of 13–18 years of age [2]. We also studied the effects of insulin-like growth factor 1 (IGF1), a hormone and paracrine mediator critical for growth [19] and organ function [20–22], which is invariably reduced in all forms of malnutrition and wasting, including anorexia nervosa [19]. We report that 97-day chronic dietary restriction initiated at 4 weeks of age in female BALB/c mice leads to persistent reduced body weights even after restoring food intake. We were able to predict chronological age based on the microbiota profile and detected microbiota immaturity in mice on chronic limited feeding. In mice whose food intake was restored after chronic dietary restriction, persistent low body weights were no longer associated with reduced microbiota age but we detected altered microbiota community structures with the animals showing microbiota changes typically seen in lean individuals and anorexia nervosa. These findings provide new insights into the mechanisms of persistent changes following exposure to restricted diet and offer a model for interventional studies.

## Methods

### Animal studies

**Fig. 1:**
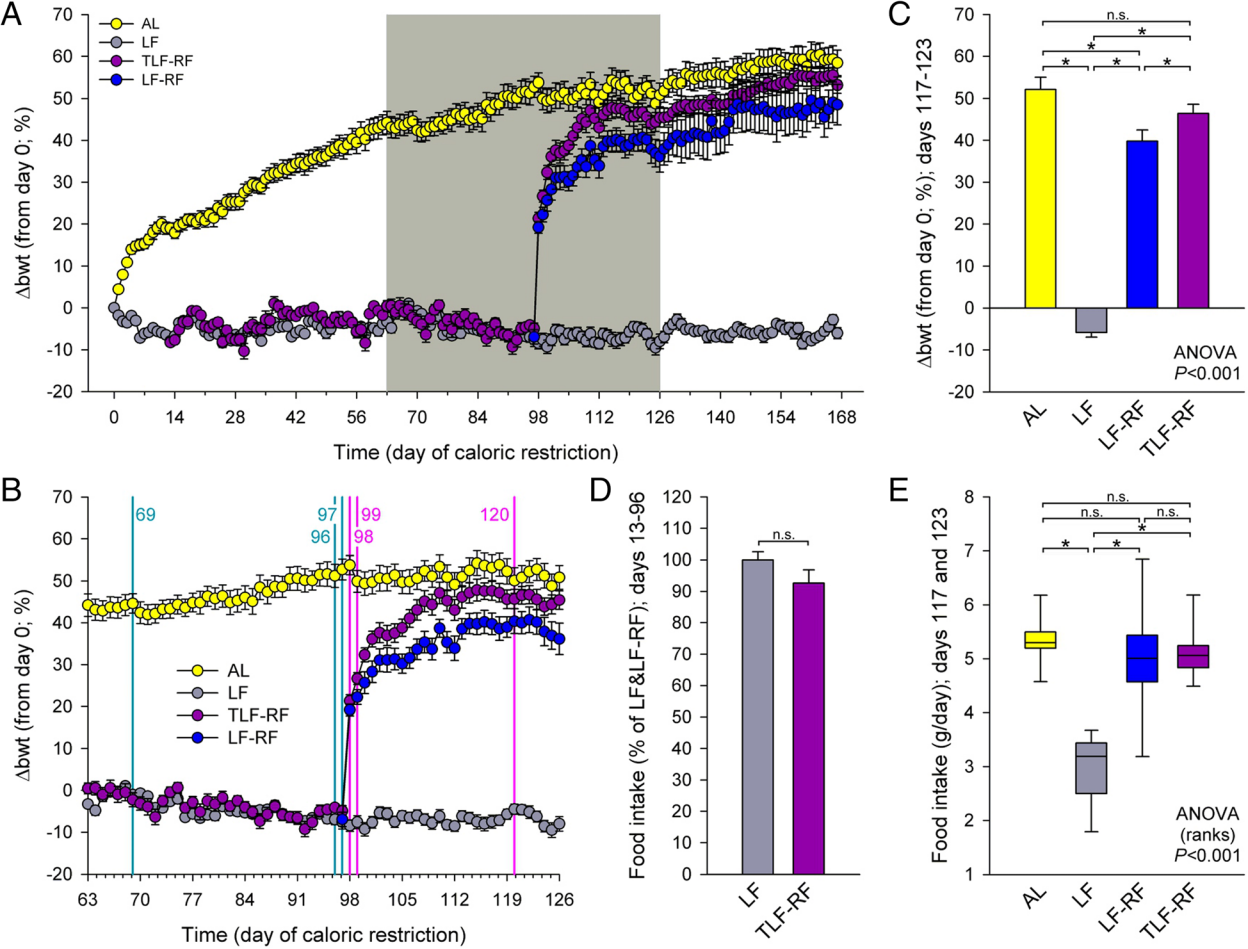
Low body weights persist following correction of chronic dietary restriction initiated at post-weaning in female mice. **a** Time course of normalized body weight changes from day 0 (Δbwt) representing 4 weeks of age (n = 12 mice/group). *AL* ad-libitum-fed cohort, *LF* limited-fed mice subjected to dietary restriction titrated to prevent weight gain, *LF-RF* limited-fed-refed mice representing a subset of LF mice given unrestricted access to food following 97 days of dietary restriction, *TLF-RF* a subset of LF-RF mice treated with twice daily subcutaneous injections of LONG R^3^ recombinant human insulin-like growth factor 1 (LONG R^3^ rhIGF1), a potent IGF1 analog with reduced affinity for IGF-binding proteins, from day 13 of the study. **b** Time period identified by *gray shading* in A. *Vertical lines* indicate feces collection. **c** One-week average body weight changes centered on the day of the last feces collection (days 117–123). *, *P* < 0.05 by Student-Newman-Keuls multiple comparison tests. *n.s.* not significant. **d** Average food intake of TLF-RF mice between days 0 and 96 expressed as the percentage of average food intake of LF mice over the same period. *n.s.* not significant. **e** Two-day average food intake determined on days 117 and 123. *, *P* < 0.05 by Dunn’s multiple comparison tests. *n.s.* not significant. LF mice weighed ~60 % less than AL controls after 167 days of dietary restriction. Body weights did not recover for at least 10 weeks after ad libitum refeeding despite comparable food intake. LONG R^3^ rhIGF1 facilitated body weight recovery

Experiments were performed in accordance with the National Institutes of Health Guide for the Care and Use of Laboratory Animals. All protocols were approved by the Institutional Animal Care and Use Committee of the Mayo Clinic (A65814). Forty-eight female BALB/c mice were purchased from Harlan Laboratories (Madison, WI, USA). Female mice were used because female-to-male ratios in anorexia nervosa range from 6:1 to 10:1 [2]. Upon receipt at 3 weeks of age, all mice received a ten-digit Pro ID radiofrequency identification chip (Microchip ID Systems, Covington, LA, USA) injected subcutaneously at the nape of the neck. The animals were randomized into four equal groups and housed individually in cages fitted with white cage lining paper to facilitate recovery of uneaten food for food intake measurements. Body weight was measured daily between 08:00 and 10:00 before feeding with LabDiet (St. Louis, MO, USA) 5053 PicoLab® Rodent Diet 20 (irradiated; protein: 21 %, fat: 5 %, crude fiber: 4.6 %, nitrogen-free extract: 53.4 %, gross energy: 4.11 kcal/g; ash: 5.9 %). At 4 weeks of age, i.e. close to the age mice achieve sexual maturity, which was designated as study day 0, the groups were rebalanced by weight to minimize inter-group variability that developed during the week of acclimatization (mean ± standard deviation: Group 1: 15.65 ± 1.09 g, Group 2: 16.16 ± 0.98 g, Group 3: 15.79 ± 1.12 g, Group 4: 16.02 ± 0.74 g; analysis of variance (ANOVA) *P* = 0.592; n = 12/group). Group 1 continued to receive ad libitum feeding throughout the study (AL group; Table 1, Fig. 1). Groups 2–4 started receiving individualized rations of the same mouse chow adjusted daily based on body weights measured before feeding to prevent natural weight gain and keep normalized body weight change from day 0 (Δbwt) between 0 and −10 % (dietary restriction by limited chow feeding). A similar approach has been shown to increase the life span of mice and protect brain neurons from excitotoxic stress [23]. Mice tolerated this regimen well and consumed their rations within ~3 h. Group 2 was maintained on this regimen throughout the study (limited-fed group, LF). IGF1 levels are reduced in all forms of protein-energy malnutritions including anorexia nervosa [19] and reduced IGF1 expression was also detected in mice fed a malnourished diet (7 % protein and 5 % fat) for 21 days starting at weaning [24]. In preliminary studies performed in a separate cohort of mice, we also detected reduced serum IGF1 levels in LF mice relative to AL controls (mean ± standard deviation: 236 ± 75 ng/mL, n = 12 versus 361 ± 136 ng/mL, n = 8; *P* = 0.016). Therefore, on day 13, following stabilization of body weights, Group 4 mice (on limited feeding) started receiving twice daily subcutaneous injections of LONG R^3^ recombinant human IGF1 (a potent IGF1 analog with reduced affinity for IGF-binding proteins; Research Peptides, Orlando, FL, USA; 150 μg/kg [21]) to facilitate body weight gain. We started IGF1 treatment during the limited feeding phase because in preliminary studies this preconditioning paradigm achieved greater body weight gain than treatment initiated at the time of refeeding. On day 97, following body weight measurement, mice in Groups 3 and 4 were restored to ad libitum feeding (limited-fed-refed, LF-RF, and IGF1-treated limited-fed-refed, TLF-RF, mice, respectively). On days 117 and 123, daily food intake was determined by weighing the chow offered and the amount recovered from the cages 24 h later (Fig. 1e). The latter was accomplished by drying all cage contents to weight constancy under a heat lamp and removing fecal pellets and pieces of cage lining paper manually. Average intake/day was calculated for each mouse from the day 117 and day 123 data. For the microbiome studies, fecal pellets were collected into sterile tubes placed under the anus on days 69, 96, 97, 98, 99, and 120 (Fig. 1b) immediately before feeding at 10:00. Pellets were collected for 2 days immediately before and after refeeding (days 96 and 97 and days 98 and 99, respectively) to assess potential rapid changes occurring in response to lifting dietary restriction.

**Table 1:** Overview of experimental groups

Group No.	Group name	Limited feeding (study days)	Ad libitum feeding (study days)	LONG R^3^ rhIGF1^a^ (study days)
1	AL	—	0–167	—
2	LF	0–167	—	—
3	LF-RF	0–96	97–167	—
4	TLF-RF	0–96	97–167	13–167

^a^LONG R^3^ recombinant human IGF1; 2 × 150 μg kg^−1^ day^−1^ subcutaneously

### 16S rRNA gene amplicon preparation, sequencing, and processing

DNA extraction was carried out according to the Manual of Procedures on the Human Microbiome Project website (http://www.hmpdacc.org/) using physical and chemical lysis with a FastPrep-24 (MP Biomedicals, Santa Ana, CA, USA) and PowerSoil Extraction Kit (MoBio, Carlsbad, CA, USA). Amplification targeted the V3-V5 hypervariable region of the gene encoding the bacterial 16S subunit of ribosomal RNA using primers 357 F (AATGATACGGCGACCACCGAGATCTACACTATGGTAATTGTCCTACGGGAGGCAGCAG) and 926R (CAAGCAGAAGACGGCATACGAGAT-NNNNNNNNNNNN-AGTCAGTCAGCCCCGTCAATTCMTTTRAGT) with barcodes 1–40 incorporated in the reverse primer. PCR was run through 34 cycles of 98 °C for 15 s, 70 °C for 20 s, and 72 °C for 15 s with Kapa HotStart HiFi DNA Polymerase (Kapa Biosystems, Boston, MA, USA). Electrophoresis of a small sample was used to verify amplicon specificity and purification was carried out using magnetic beads. DNA for each amplicon was then diluted to 10 nM and pooled for sequencing on a MiSeq instrument (Illumina, San Diego, CA, USA) using a 300 cycle kit and custom read1 (TATGGTAATTGTCCTACGGGAGGCAGCAG), read2 (AGTCAGTCAGCCCCGTCAATTCMTTTRAGT), and index (ACTYAAAKGAATTGACGGGGCTGACTGACT) sequencing primers [25]. This produced non-overlapping reads, which were then analyzed by the IM-TORNADO and *mothur* pipeline using default parameters [26, 27] (see details in Additional file 1).

### Statistical analysis

We summarized microbiota data using both alpha diversity and beta diversity. Alpha diversity reflects species richness and evenness within bacterial populations. Beta diversity reflects the shared diversity between bacterial populations in terms of ecological distance; different distance metrics provide distinct views of community structure. Four alpha-diversity measures (observed OTU number, Chao 1 estimator, Shannon index, and inverse Simpson index) and three beta diversity measures (unweighted, generalized (α = 0.5), and weighted UniFrac distances) were calculated to obtain a comprehensive view of the microbiota [28]. Linear mixed effects model was used to test for covariate effect on alpha diversities. A random intercept was included for each subject to account for within-mouse correlation. PERMANOVA was used to test for association of covariates with the beta diversities [29]. To account for within-mouse correlation, permutation was constrained within each subject if necessary. The distance-based R^2^ from PERMANOVA was used to quantify the relative contribution of age, diet, and Δbwt to the microbiota variability. Principal component analysis (PCA) based on unweighted UniFrac distance matrix was used for visualizing sample relationships. To identify microbial taxa showing dependence on their pre-refeeding state, overdispersed Poisson regression model was used to fit the observed taxon counts. False discovery rate (FDR) control based on Benjamini–Hochberg procedure [30] was performed to correct for multiple testing. An adjusted *P* or *Q* < 0.1 was considered statistically significant. For all analyses, covariates were adjusted if necessary. The machine learning algorithm Random Forests [31] was used to predict the age based on the microbiota profile (OTU level) using default parameters of the R implementation of the algorithm. OTUs with a prevalence less than 10 % and proportion less than 0.1 % in all samples were excluded. Bootstrapping (500 bootstrap samples) was used to assess the prediction accuracy. The prediction mean squared error (PMSE) was compared to the best guess and Friedman Rank Sum test was used for testing the significance of the difference. The Boruta feature selection algorithm, which wraps around Random Forests [32], was applied for selecting the age-discriminatory taxa. Further technical details can be found in Additional file 1. All statistical analyses were performed in R-3.0.2 (R Development Core Teams).

## Results

### Low body weights persist following correction of chronic dietary restriction in young female mice

During the 24-week study, Δbwt of AL mice increased logarithmically (R^2^ = 0.9853 when regressed to f = if(x-x_0_ > 0, y_0_ + a*ln(abs(x-x_0_)), 0) (Fig. 1a). Δbwt of LF mice could be kept within pre-set limits by feeding them daily titrated amounts of mouse chow representing 56 ± 12 % (mean ± standard deviation) of daily AL intake, which they tolerated well and consumed within ~3 h. The average amount of food offered to LF mice was 2.97 ± 0.42 g and changed very little throughout the study (slope from linear regression; days 0–167: –0.0003). LF mice remained healthy, vigorous, and active throughout the study despite weighing on average 58 % less than AL mice during the 1-week period centered on the last feces collection on day 120 (Fig. 1c). During the period of limited feeding (days 0–96), LF-RF mice received 2.86 ± 0.24 g food. TLF-RF mice had to be offered slightly less food than untreated restricted mice due likely to the anabolic effect of the IGF1 analog (2.73 ± 0.36 g; 92.6 ± 14.6 % of all LF mice; *P* = 0.117; Fig. 1d). Upon restoration of ad libitum feeding on day 97, Δbwt of LF-RF mice increased rapidly for 12 days before assuming a time-course paralleling AL values approximately 12 % below normalized AL weights, a significant difference maintained to the end of the study (days 117–123; Fig. 1a–c). Δbwt of TLF-RF mice followed a similar time course but their normalized weights were within 6 % of, and not significantly different from, AL weights. TLF-RF Δbwt values were, however, significantly higher than LF-RF Δbwt values at the end of the study (Fig. 1c). With the exception of the LF group, whose food intake was titrated to prevent body weight gain, all mice in all groups continued growing throughout the study. The body weight differentials observed at the end of the study occurred in the absence of significant differences in average food intake among AL, LF-RF, and TLF-RF mice calculated from the intake measured on days 117 and 123 (Fig. 1e). These results indicate that chronic dietary restriction initiated at 4 weeks of age in female mice leads to persistent reduced body weights even when dietary restriction is lifted. This can be corrected by treatment with a potent analog of the anabolic hormone IGF1, which is invariably reduced in caloric restrictions [19].

### Age and diet but not body weight change associate with gut microbiota composition

**Fig. 2:**
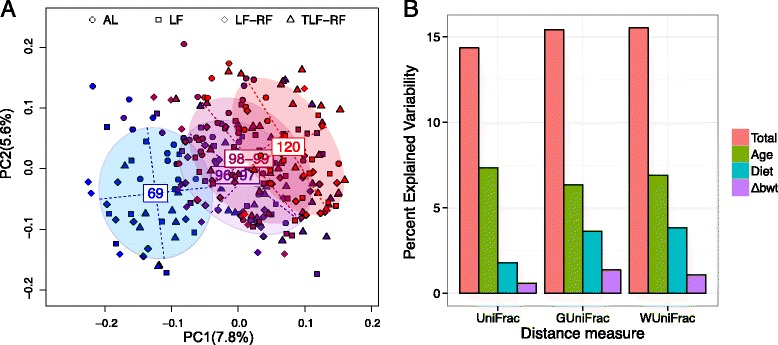
Age explains more microbiota variability than diet and body weight. **a** The first two PCs from the PCA on the unweighted UniFrac distance matrix are *plotted. Symbols* represent data from individual diet regimens color-coded by sampling days. The main axes of the ellipses correspond to the PCs of the group with the heights and widths representing variances in the corresponding components. **b** The percentage of microbiota variability explained by age, diet type, Δbwt, and their combination (total) based on different UniFrac distances. UniFrac, GUniFrac, and WUniFrac represent unweighted, generalized (α = 0.5), and weighted UniFrac distance, respectively. Non-linear age effects are assumed

To investigate the relationship between gut microbiota and the persistence of reduced body weights after the restoration of ad libitum food access following chronic dietary restriction, we first studied the potential associations between the mouse gut microbiota composition and host factors such as age, diet type, and body weight change. We performed distance-based multivariate analysis (PERMANOVA [29]) using fecal samples from the AL and LF diet groups and included Δbwt, age, and diet type as covariates and the gut microbiota composition, which was summarized using UniFrac distance matrices as the outcome variable. To account for potential non-linear age effects, we treated age as a categorical variable by combining experimental days 96–99 into one group since they were consecutive and showed no significant differences (Additional file 2A, B). Age and diet were significantly associated with the gut microbiota composition adjusting for other factors (*P* < 0.05 for all distance metrics, Table 2). Δbwt was not significantly associated with the microbiota composition after adjusting for age and diet effects (*P* > 0.05). PCA using unweighted UniFrac distance revealed that age effects could be captured by the first principal component (PC) (Fig. 2a) and the effects were consistent across all four diet groups (Additional file 2). The alpha diversity of the gut microbiota also increased with age (Additional file 3). The LF group had significant increase in all four alpha diversity measures investigated (*P* = 1.9e-10, 2.3e-7, 1.4e-10, and 2.8e-9, respectively) while the AL group increased only in species richness (*P* = 0.007 and 0.0008 for observed number of OTUs and Chao1 estimator, respectively) but not in overall diversity (*P* = 0.68 and 0.93 for Shannon and inverse Simpson diversity indices, respectively).

**Table 2:** Relative contribution of age, diet, and weight to the overall microbiota variability^a^

Distance^b^	Age		Diet		Weight		Total	
	R^2 c^	*P* value^d^	R^2^	*P* value	R^2^	*P* value	R^2^	*P* value
UniFrac	7.3 %	<0.001	1.8 %	<0.001	0.6 %	0.429	14.4 %	<0.001
GUniFrac	6.4 %	<0.001	3.6 %	<0.001	1.4 %	0.167	15.4 %	<0.001
WUniFrac	6.9 %	0.003	3.8 %	0.008	1.1 %	0.139	15.5 %	<0.001

^a^Based on ad libitum-fed (AL) and limited-fed (LF) diet groups

^b^UniFrac, GUniFrac, and WUniFrac represent unweighted, generalized (α = 0.5), and weighted UniFrac distance, respectively

^c^R^2^ represents the percentage of microbiota variability explained by corresponding covariate adjusting for other covariates

^d^Significance was assessed based on 1000 permutations. For age and weight, permutation was confined within each subject

We next quantified the relative contribution of diet, age, and Δbwt to the overall microbiota variability. Different UniFrac distances were used to summarize the overall microbiota variability and the variability was partitioned among different sources (age, diet type, and Δbwt) using PERMANOVA. Assuming non-linear age effects, the proportion of overall variability explained by the three factors was about 15 % for all the three UniFrac distances (Fig. 2b, Table 2). Therefore, there was significant inter-animal variability that could not be explained by the host or dietary factors, consistent with previous findings [33]. Age explained a larger proportion of the observed microbiota variability (6.4–7.3 %) than diet (1.8–3.8 %) and body weight (0.6–1.4 %). Relative to diet and body weight, the explanatory power of age was much larger when unweighted UniFrac distance was used, suggesting that the community structure of gut microbiota changed significantly with age, whereas diet and body weight were more associated with species abundance change. Collectively, these results indicate that of the host factors examined, age, diet type, and Δbwt contribute in a decreasing order to the diversity and variability of the gut microbiota in mice.

### Chronic dietary restriction is associated with relative microbiota immaturity

**Fig. 3:**
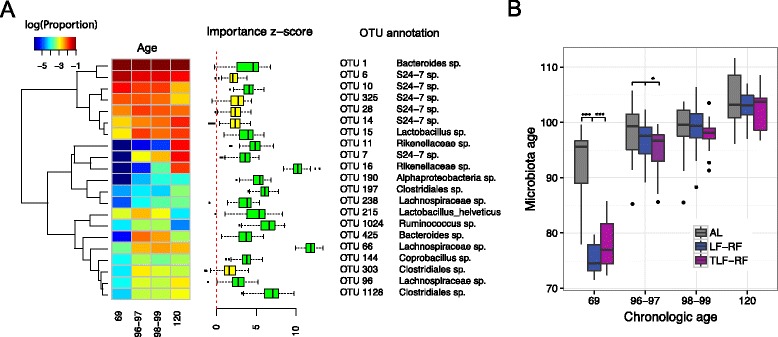
Predicting mouse chronologic age based on gut microbiota using Random Forests. **a** *Heat map* of the mean relative abundance of age-discriminatory OTUs selected by the Boruta algorithm for the AL diet group. *Rows* represent the OTUs and *columns* represent the sampling day (Age). *Hierarchical clustering* on the *left* was based on complete linkage and Euclidean distance. Importance Z-scores from the Boruta alogrithm are *plotted* on the *right*. A large importance Z-score indicates stronger ability of corresponding OTU to discriminate chronological age. *Green* and *yellow colors* indicate the significance level (‘confirmed’ and ‘tentative’, respectively). **b** Predicting the age of the microbiota samples from the other diet groups using samples from the LF group as the training set. The *y axis* represents the predicted age (microbiota age) by Random Forests. *Colors* represent individual diet groups. Mice under dietary restriction (LF-RF and TLF-RF groups before the reintroduction of the ad libitum diet) exhibited lower microbiota ages than AL mice

Recent studies in children with severe or moderate acute malnutrition described gut microbiota immaturity [7, 13] that was only partially ameliorated by therapeutic interventions paralleling incomplete restoration of healthy growth [7]. To investigate whether a similar phenomenon is present in our mouse model, we used Random Forests [31] to build an age-predictive model using the OTU-level relative abundance data. Random Forests achieved a much lower PMSE than non-informative guess (*P* < 2.2E-16, Friedman Rank Sum test, Additional file 4). To identify the OTUs that contributed significantly to the prediction performance, we applied the Boruta feature selection algorithm [32]. The Boruta algorithm selected a total of 21 significant OTUs (Fig. 3a). Six OTUs came from the uncultured family *S24*-7 from the phylum *Bacteroidetes* and the rest mainly from the order *Clostridiales* (*Ruminococcaceae*, *Lachnospiraceae*, and *Clostridiaceae*). OTU 16 and OTU 66 from the *Rikenellaceae* and *Lachnospriaceace* families had the strongest discriminatory power. To test whether the gut microbiota profile had sufficient age-predictive power in our sample sets, we trained the model using samples from the AL group and predicted the age of the samples from the other diet groups. The model achieved good age-discriminatory performance across all the other diet groups (R^2^ = 0.70, 0.82, and 0.69 for LF, LF-RF, and TLF-RF, respectively (Additional file 4).

To investigate whether the microbiota under chronic dietary restriction is relatively immature, we next built an age-predictive model using the LF group as a training dataset and then predicted the age of the samples from the other three groups. This approach was necessitated by all groups other than the AL group receiving limited feeding between days 69 and 97, requiring one of them to be used as training set to assess their microbiota age relative to the ad libitum-fed mice. Indeed, the AL group was predicted to have a much more advanced microbiota age on day 69 (Fig. 3b, *P* < 0.001, *t* test), indicating relative immaturity of the gut microbiota under chronic dietary restriction. However, upon refeeding, i.e. when the diet of the LF-RF and TLF-RF groups switched to the ad libitum diet, the difference was no longer significant suggesting that the diet change could move the gut microbiota towards the ad libitum state (Fig. 3b). These results indicate that gut microbiota immaturity described in children with severe acute malnutrition [7, 13] is demonstrable in our clinically much more benign chronic dietary restriction model; however, ad libitum refeeding was able to correct for this change despite persistent reduced body weights seen in the LF-RF group.

### Altered gut microbiota community structure persists after correction of chronic dietary restriction

**Fig. 4:**
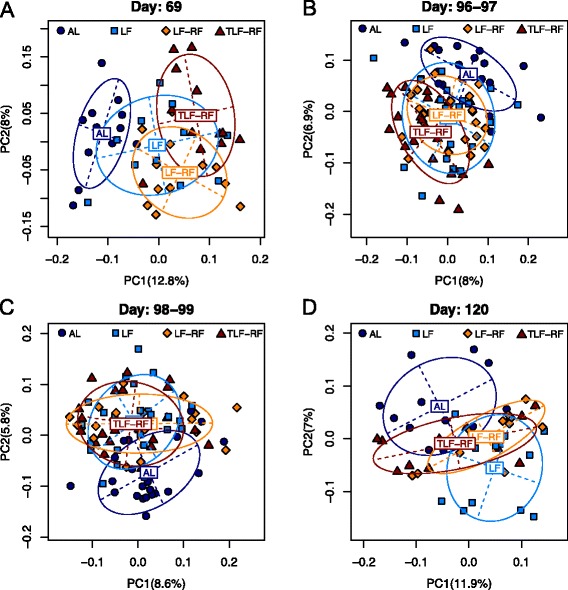
Altered gut microbiota community structure persists after correction of chronic dietary restriction. **a**–**d** PCA on days 69 (**a**), 96–97 (**b**), 98–99 (**c**), and 120 (**d**). The first two PCs from the PCA on unweighted UniFrac distance matrix are plotted. *Symbols* and *colors* represent data from individual diet regimens. The main axes of the ellipses correspond to the first two PCs with the height and width representing variances in the corresponding coordinates. Note that the LF-RF and TLF-RF data remained different from the AL data on day 120 despite a significant separation from the LF group; and that IGF1 treatment (TLF-RF group) mitigated the difference from the AL mice

To identify additional measures that would reflect persistent reduced body weights seen in our model after refeeding, we next investigated in greater detail the changes in gut microbiota community structures with time under different diet regimens. As expected, no significant difference in community structure was detected in the gut microbiota of the LF and LF-RF mice on day 69 (*P* = 0.19, PERMANOVA test, unweighted UniFrac), when both groups were on the limited diet (Fig. 4a). In contrast, the gut microbiota of AL mice was significantly different from the gut microbiota of mice on the restricted diet (LF and LF-RF groups; *P* < 0.001), indicating that limited feeding had significant effects on the gut microbiota structure (Fig. 4a). IGF1 treatment of limited-fed mice (TLF-RF group) also had significant effects on the gut microbiota (*P* = 0.002, Fig. 4a). The same trends were detected on days 96–97, i.e. immediately before refeeding (Fig. 4b). On days 98–99, i.e. immediately after switching to the ad libitum diet, the microbiota structure of LF-RF mice was still significantly different from that of the AL group (*P* < 0.001) and much closer to the gut microbiota of the LF group (*P* = 0.029, Fig. 4c). This suggests short-term resilience of the microbiota in response to diet change. On day 120, i.e. 3 weeks after refeeding, the LF-RF group was significantly different from both the LF group (*P* = 0.002) and the AL group (*P* < 0.001) indicating that the microbiota changed in response to refeeding but did not assume the state of the AL group. The microbiota of the TLF-RF group was also different from both AL and LF group (*P* = 0.01 and 0.003, respectively) but the difference from the AL group was smaller as indicated by a shorter between-group distance (*P* = 0.078). These results paralleled the improved weight recovery in response to the anabolic preconditioning and treatment during refeeding. These findings indicate hysteresis effect on the gut microbiota, i.e. a dependence of the microbiota structure on their state at the initiation of refeeding (Fig. 4d).

**Fig. 5:**
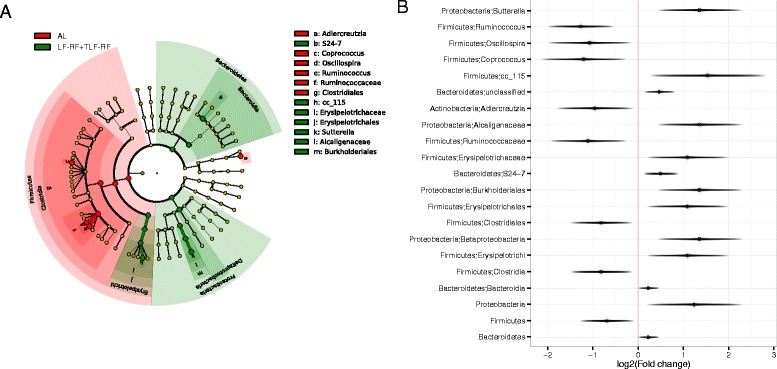
Specific bacterial taxa show hysteresis effect under chronic dietary restriction. **a** *Cladogram* generated with GraPhlAn (http://huttenhower.sph.harvard.edu/galaxy/) showing “hysteresis” bacterial taxa identified by comparing their abundance in the AL group to LF-RF and TLF-RF mice (refed groups) on day 120. *Red* represents abundance increase in the AL group and *green* represents abundance increase in the refed groups. **b** Log_2_ fold change (refed groups/AL) of the abundance of taxa identified at an FDR of 10 %. The *horizontal fuzzy line* represents the 95 % confidence interval of the log fold change estimate

To identify microbial taxa showing significant differences between the AL group and the refed groups LF-RF and TLF-RF, we performed differential abundance analysis using day 120 samples including diet type, refeeding status, and IGF1 treatment status as covariates. At a FDR of 10 %, we identified 21 significant taxa at different taxonomical levels (Fig. 5a). The fold changes of these taxa were in the range of 1.14–2.80 (Fig. 5b). The refed groups had a higher abundance of *Bacteroidetes* and *Proteobacteria* and a lower abundance of *Firmicutes* at the phylum level. The abundance of the genera *Ruminoccocus*, *Oscillospria*, *Coprococcus*, and *Adlercreutzia* was decreased and the abundance of *Sutterella* and *cc_115* (Firmicutes) was increased in the refed groups. Although by using the same approach we could only detect relatively weak association between microbiota and Δbwt on day 120 (Additional file 5), the most significant genus, *Adlercreutzia* (*P* = 4E-5), which was associated with higher body weights, was also significantly underrepresented in the refed groups. Together, these results indicate that persistence of lower body weights in the refed groups (particularly in LF-RF mice) occurring in the absence of significantly lower food intake (Fig. 1a–e) was associated with microbiota dominating the gut flora in lean mice and humans and patients with anorexia nervosa [9, 11, 12, 14–16].

## Discussion

Restoration of body weights and prevention of growth failure in childhood and adolescent malnutrition or wasting diseases including anorexia nervosa have been challenging as the mechanisms underlying the failure of nutritional interventions remain unclear. Recent studies have demonstrated microbiota alterations persisting in malnourished children and anorexia nervosa patients with transient and incomplete responses to nutritional therapy [7, 9, 13–16]. Gut microbiota are also altered in diseases that cause wasting in children, such as chronic kidney and liver disease, short bowel syndrome, inflammatory bowel disease, and HIV infection [34–39]. Dysbiosis has been shown to play a causal role in undernutrition and its consequences [13]. However, further in-depth investigation will require animal models that reproduce the pathophysiology of dietary restrictions and complement approaches involving microbiota transfer into germ-free mice, which differ from conventionally raised mice in physiological parameters, e.g. gastrointestinal motility [18]. Here we report that chronic dietary restriction initiated at 4 weeks of age in female mice and titrated daily to prevent body weight gain is a suitable model of growth failure occurring in malnourished humans despite nutritional interventions because it led to persistent reduced body weights even after restoration of age-appropriate and sex-appropriate food intake. This model is most closely related to anorexia nervosa, where self-imposed dietary restriction or other weight loss behaviors lead to severe emaciation in the presence of hunger, mainly in young females [9, 40]. Body weight recovery could be facilitated by treatment with a potent analog of the anabolic hormone IGF1, which is invariably reduced in dietary restrictions including anorexia nervosa [19]. In our model, age and diet type, but not Δbwt, were associated with gut microbiota composition; and age explained a larger proportion of the microbiota variability than diet or Δbwt. We could only demonstrate microbiota immaturity during the period of restricted feeding. However, altered microbiota community structures persisted even after ad libitum refeeding indicating that similarly to humans, microbiota changes are associated with incomplete responses to nutritional rehabilitation in mice. Interestingly, the IGF1 treatment also mitigated the microbiota changes suggesting an important role for host factors in this paradigm, possibly via regulation of gastrointestinal motility [22]. However, our design, which did not include IGF1 treatment of ad libitum-fed mice, did not permit answering the question whether the IGF1 effects on the microbiota were directly related to weight restoration following chronic dietary restriction or reflected nutritional state-independent actions. Analysis of the microbial taxa accounting for the hysteresis effect revealed changes in microbiota composition involving a shift toward bacterial taxa dominating gut flora of lean mice and humans, which is consistent with the observed lower body weights occurring in the absence of lower food intake.

Interestingly, age had a much larger effect on the mouse gut microbiota than diet and samples tended to cluster by age rather than diet type. However, consistent with a previous report indicating reduced day-to-day variability in mice after 11 days post-weaning [41], age still only captured ~7 % of the overall microbiota variability. Alpha diversity of the gut microbiota tended to increase with age. Δbwt was not significantly associated with the overall gut microbiota after adjusting for age effects. The relatively large age effects on the gut microbiota prompted us to build up an age-predictive model based on the microbiota profile [7, 13]. We used the Random Forests algorithm to predict the chronological age based on the microbiota profile and achieved good prediction performance. Thus, consistent with a previous human studies on the gut microbiota in malnourished children [7, 13], the mouse gut microbiota under chronic dietary restriction exhibited relative immaturity. We also found hysteresis effect on the mouse gut microbiota, where the gut microbiota under chronic dietary restriction could not return to the ad libitum-fed state even after a prolonged period of ad libitum diet. These findings indicate the utility of our model as an experimental tool that reproduces host physiology and pathophysiology to study the role of microbiota in malnutrition using interventional approaches, which would not be practical or ethical to perform in malnourished human subjects.

Recently published alternative models of early-life human malnutrition involving conventionally raised mice differ from ours in some key areas. Post-weaning mice fed a calorically compensated low-protein diet (7 % protein and 15 % fat) were shown to display features of human environmental enteropathy, a major contributor to childhood malnutrition, along with a remodeling of gut bacterial communities [24]. A notable difference between this and our model is that mice in our protocol received reduced but unaltered food, which they consumed within ~3 h. Overall reduced intake of energy and all major nutrients is characteristic of the diet of patients with anorexia nervosa [42], whereas malnutrition dominated by reduced protein intake is more typical in children living under low socioeconomic conditions [24]. Another recently introduced model of human undernutrition involves timed separation of neonatal mice from lactating dams [43, 44]. In these mice, the altered microbiota and microbial metabolites persisted despite catch-up growth following refeeding [44]. Similar to our protocol, limited suckling is characterized by overall reduced food intake but with exposure to restricted diet occurring at a younger age. Thus, our approach complements previously established models by focusing on malnutritions that begin around the attainment of sexual maturity and dominated by overall reduced energy intake.

Large age and diet effects on the gut microbiota had been observed in many studies [7, 45–47]. The balanced design of the study enabled us to dissect the relative contribution of host factors including age, diet type, and Δbwt to the overall microbiota variability using a multivariate regression model based on distance metrics. We found that the total variability in mouse gut microbiota that could be explained by age, diet type, and Δbwt was around 15 % using different UniFrac distance metrics. Clearly, there was a significant amount of variability that could not be explained by these host factors, which could be due to inter-subject and inter-day variability or even measurement error.

The Random Forests algorithm had been successfully applied in various supervised learning tasks based on microbiota profile [7, 48–50]. When we trained the Random Forests algorithm using samples from the LF group and predicted the age for the samples from other groups, we did not see significant difference of the predicted age between the AL and LF-RF or TLF-RF groups on day 120, when we expected to see hysteresis effects. This might reflect the more benign dietary restriction the mice in our study experienced than seen in clinical malnutrition. However, this approach was able to detect microbiota immaturity during the period of dietary restriction and had good age-discriminatory power. Therefore, it appears more likely that the lack of significant difference between the AL and refed groups was due to the limited age resolution of the predictive model trained on only four time points and a relatively small sample size, which led to suboptimal match between the predicted and chronological ages and a failure to distinguish a subtle effect such as the hysteresis effect.

While we could not detect microbiota immaturity in association with persistent low body weights—which probably reflected the limitations of our approach—gut microbiota was not restored by refeeding after chronic dietary restriction lasting more than 3 months. Analysis of the contributing taxa indicated higher abundance of *Bacteroidetes* and *Proteobacteria* and lower abundance of *Firmicutes* in the refed groups. Underrepresentation of *Adlercreutzia* was also significant in relation to Δbwt. Higher *Bacteroidetes*-to-*Firmicutes* ratios have been found in lean mice and humans and anorexia nervosa patients, and transplantation experiments have demonstrated the role of these changes in conferring lower body weights on obese recipients [11, 12, 14–16]. Similar changes were detected in the duodenum of post-weaning mice fed a malnourished diet [24]. In contrast, *Bacteroides* or *Bacteroidetes* are reduced in acute-on-chronic liver failure, short bowel syndrome, inflammatory bowel disease, and HIV infection with variable changes in *Firmicutes* [35–38]. Furthermore, a reduced *Bacteroidetes*-to-*Firmicutes* ratio was observed upon complete body weight recovery in response to refeeding following the timed separation of neonatal mice from lactating dams [44]. Thus, the association of microbiota dominating the gut flora in lean mice and humans with persistence of lower body weights in the refed groups in our study suggests that the observed microbiota changes could be mediators of the reduced body weight gains in mice with long-term caloric restriction started at a young age. However, future studies involving experimental manipulation of gut microbiota will be required to prove a causal relationship in our model. Future studies will also determine whether this model of chronic dietary restriction followed by ad libitum refeeding also results in persistent low body weights and corresponding alterations in gut microbiota in male mice.

## Conclusions

Dietary restriction by limited chow feeding initiated at 4 weeks of age in female mice and maintained for more than 3 months led to persistent growth failure following the restoration of ad libitum food access. In this model, persistent low body weights were associated with changes in microbiota composition involving a shift toward bacterial taxa dominating gut flora of lean mice and humans. These alterations occurred against the backdrop of large age-related shifts in microbiota composition. Our results provide new insights into the mechanisms of persistent changes following chronic dietary restriction and offer a physiologically relevant model for interventional studies designed to improve responses to nutritional therapy and prevent growth failure in wasting diseases and malnutrition of various etiologies including anorexia nervosa.

## Additional files

Additional file 1: Supplementary Methods. (PDF 686 kb) Additional file 2: The age effect on gut microbiota is consistent across diet groups. The first two PCs from the PCA on the unweighted UniFrac distance matrix are plotted. **A**–**D** *Samples* from different diet groups. Samples are *color-coded* by sampling days. The main axes of the ellipses correspond to the principal components of the group with the heights and widths representing variances in the corresponding components. (PDF 536 kb) Additional file 3: Alpha diversity of the gut microbiota increases with age. OTU data were rarefied before calculating the diversity measures. Four diversity measures are shown: observed number of OTUs (Observed), Chao1 species richness estimator (Chao1), Shannon diversity index (Shannon), and inverse Simpson diversity index (InvSimpson). All diversity measures increased in LF, LF-RF, and TLF-RF mice, whereas only species richness indices (Observed and Chao1) increased in AL mice. (PDF 787 kb) Additional file 4: Gut microbiota have strong age-predictive power. **A** Random Forests analysis achieves much smaller PMSE than the prediction based on the mean age in the training set (Guess). **B** The age of the microbiota samples was predicted using samples from AL diet group as the training set. The *y axis* represents the predicted age (microbiota age) by Random Forests analysis. *Colors* represent individual diet groups. (PDF 136 kb) Additional file 5: Log_2_ fold change of the abundance of body weight-associated taxa in response to one unit change of body weights. (PDF 9 kb)

## Abbreviations

AL: Ad libitum*-*fed group
ANOVA: Analysis of variance
FDR: False discovery rate
IGF1: Insulin-like growth factor 1
LF: Limited-fed group
LF-RF: Limited-fed-refed group
OTU: Operational taxonomic unit
PERMANOVA: Permutational multivariate analysis of variance
PMSF: Prediction mean squared error
TLF-RF: Treated limited-fed-refed group
Δbwt: Normalized body weight change from day 0
