# Supplementary material for: Altered gut microbiota in female mice with persistent low body weights following removal of post-weaning chronic dietary restriction
Source: Genome Med. 2016 Oct 3;8:103. doi: 10.1186/s13073-016-0357-1 (PMC5048651; doi:10.1186/s13073-016-0357-1)
Supplement: Additional file 1: — Supplementary Methods. (PDF 686 kb) [file 13073_2016_357_MOESM1_ESM.pdf]

Supplementary Methods

Pipeline for processing of 16S rRNA gene sequencing data. We used the IM-TORNADO pipeline to process the 16S rRNA gene sequencing data [1]. Pre-processed sequence files were subjected to quality filtering using Trimmomatic version 0.22 [2], with a hard cut-off of PHRED score Q3 for the 5' and 3' ends of the reads (parameters LEADING:3 and TRAILING:3), trimming of the 3' end with a moving average score of Q15, window size: 4 bases (parameter SLIDINGWINDOW:4:15), and removing any remaining reads shorter than 75% of the original read length (parameter MINLEN:112 for reads of 150 bp). Reads with any ambiguous base calls or with homopolymers longer than 10 bases were discarded using *mothur* [3]. Only the read pairs that survived the quality filter were processed further. Any surviving unpaired reads (i.e., reads that lost their matching pair due to low quality) were discarded. Remaining read pairs were then grouped into two files, one each for "read 1" and "read 2" sequences. Reads were also de-replicated, consolidating identical reads to avoid redundant processing. Each of the two read libraries was then checked for chimeras using UCHIME in *de-novo* mode [4].

Clustering, representative sequences, and chimera removal. Paired-end reads were concatenated directly with no padding and de-replicated. Operational taxonomic unit (OTU) representatives were selected and used to generate a reference set for clustering using UPARSE [5], which was run with default parameters that also removes chimeric reads.

Taxonomy assignment. To prepare the reads for this step, we took reads from the previous step just before the stitching procedure, removed the gaps and then stitched them with a pad sequence of "N" bases. Since by default most Bayesian classifiers use 8-mers to perform the classification, we used "NNNNNNNN" as the padding. The stitched reads were then classified using the Greengenes taxonomy (v13.5) [6].

Alpha and beta diversity analysis. The alpha diversity measures such as observed number of species, Chao 1 estimator, Shannon and inverse Simpson diversity indices were calculated based on the rarefied OTU table to address different sequencing depths (*estimate_richness* function in Bioconductor package *PhyloSeq* [7]). Linear mixed effects model was used to test for age effects on alpha diversities for different diet regimens (*lme* function in the R package *nlme*). A random intercept was included for each animal to account for within-mouse correlation. Statistical significance was assessed by likelihood ratio test. Beta diversity reflects the shared diversity between bacterial populations in terms of ecological distance; different distance metrics provide distinctive views of community structure. Unweighted, generalized and weighted UniFrac distance metrics were calculated (*GUniFrac* function in the R package *GUniFrac*). Applying different distance metrics provides insight into the nature of community change. Unweighted UniFrac is most powerful to detect the community structure and membership change while weighted UniFrac

is more efficient for detecting abundance change. Generalized UniFrac strikes a balance between the two [8]. The distance-based permutational multivariate analysis of variance (PERMANOVA) [9] method was used to test for association of covariates such as age, diet and Δbwt with the overall microbiota composition while adjusting for other covariate effects (*adonis* function in the R package *vegan*). Significance was assessed by 1,000 permutations. To account for within-mouse correlation, permutation was constrained within each animal when testing for age and weight effects. Principal coordinate analysis was performed with the unweighted UniFrac distance matrix (*cmdscale* function in R) and the first two principal components were used to generate the ordination plots.

Taxon-level analysis. To identify microbial taxa showing hysteresis effects, generalized linear model (overdispersed Poisson family, log-linear link and log(total counts) offset) was used to fit the observed taxon counts from day 120 (*glm* function in R). Diet, IGF1 treatment and refeeding status were included as covariates. Statistical significance was assessed by Wald test. False discovery rate (FDR) control based on Benjamini-Hochberg procedure [10] was performed to correct for multiple testing (*p.adjust* in R standard library). FDR control was performed on each taxonomic rank. An adjusted P or Q < 0.1 was considered statistically significant.

Predicting microbiota age using Random Forests. The machine learning algorithm Random Forests (11) was used to predict the age based on the microbiota profile (OTU level) using default parameters of the R implementation of the algorithm (R package *randomForest*). OTUs with proportion less than 0.1% in all samples were removed. The Random Forests algorithm, due to its non-parametric assumptions, was able to detect both linear and nonlinear effects and potential taxon-taxon interactions, thereby identifying taxa that jointly discriminate different periods of life. Bootstrapping (500 bootstrap samples) was used to assess the prediction accuracy. Since bootstrap is a sampling process with replacement, there are on average 63.2% unique data points in each bootstrap sample. The unused data points were used as a test data set. The prediction mean squared error (PMSE) was compared to the best guess, where the age was predicted to be the mean age of the training sample, and Friedman Rank Sum test was used for testing the significance of the difference. The Boruta feature selection algorithm [12] was applied for selecting the most discriminatory taxa based on the importance values produced by Random Forests. The importance value of a genus was calculated based on the loss of accuracy by the random permutation of the abundance profile of the genus. The Boruta algorithm used spiked-in 'shadow' taxa, which were shuffled versions of real taxa, to assess whether the importance was significant. By spiking in some 'shadow' OTUs, we could determine if the importance value of a given OTU was significant, that is, whether it was discernible from the importance value that might arise from random fluctuations.

Quantification of the relative contribution of age, diet and Δbwt to the microbiota variability. The coefficient of determination R^2 for each variable adjusting for other variables was used to quantify the relative contribution. The

$R^2(X|Z)$ [9] was defined as the extra proportion of total variability explained by adding the variable X to the model with variables Z and was calculated using the formula

$$R^2(X|Z) = \frac{\mathrm{tr}(H^{X,Z}GH^{X,Z}) - \mathrm{tr}(H^Z GH^Z)}{\mathrm{tr}(G)},$$

where $\mathrm{tr}(.)$ is the trace of a matrix, $H^{X,Z}, H^Z$ are the projection matrix into the column space of (X, Z) and Z respectively, and G is the Gower's centered matrix, which is defined as

$$G = (\mathbf{I} - \frac{\mathbf{11}'}{n})A(\mathbf{I} - \frac{\mathbf{11}'}{n}),$$

where $\mathbf{I}$ is an identity matrix, $\mathbf{1}$ is a vector of 1's and $A = (a_{ij}) = (-\frac{d_{ij}^2}{2})$ is a matrix constructed using the pair-wise distances d_{ij}. In the above formula, the total variability of the microbiota was summarized using the UniFrac distance metrics.

1. Jeraldo P, Kalari K, Chen X, Bhavsar J, Mangalam A, White B, et al. IM-TORNADO: A Tool for Comparison of 16S Reads from Paired-End Libraries. PLoS ONE. 2014;9(12):e114804.

2. Bolger AM, Lohse M, Usadel B. Trimmomatic: a flexible trimmer for Illumina sequence data. Bioinformatics. 2014;30(15):2114–20.

3. Schloss PD, Westcott SL, Ryabin T, Hall JR, Hartmann M, Hollister EB, et al. Introducing mothur: open-source, platform-independent, community-supported software for describing and comparing microbial communities. Appl Environ Microbiol. 2009;75(23):7537–41.

4. Edgar RC, Haas BJ, Clemente JC, Quince C, Knight R. UCHIME improves sensitivity and speed of chimera detection. Bioinformatics. 2011;27(16):2194–200.

5. Edgar RC. UPARSE: highly accurate OTU sequences from microbial amplicon reads. Nat Methods. 2013;10(10):996–8.

6. DeSantis TZ, Hugenholtz P, Larsen N, Rojas M, Brodie EL, Keller K, et al. Greengenes, a chimera-checked 16S rRNA gene database and workbench compatible with ARB. Appl Environ Microbiol. 2006;72(7):5069–72.

7. McMurdie PJ, Holmes S. phyloseq: an R package for reproducible interactive analysis and graphics of microbiome census data. PLoS ONE. 2013;8(4):e61217.

8. Chen J, Bittinger K, Charlson ES, Hoffmann C, Lewis J, Wu GD, et al. Associating microbiome composition with environmental covariates using generalized UniFrac distances. Bioinformatics. 2012;28(16):2106–13.

9. McArdle BH, Anderson MJ. Fitting Multivariate Models to Community Data: A Comment on Distance-Based Redundancy Analysis. Ecology. 2001;82(1):290–7.

10. Benjamini Y, Hochberg Y. Controlling the false discovery rate: a practical and powerful approach to multiple testing. J Roy Stat Soc B Met. 1995;57(1):289–300.

11. Breiman L. Random Forests. Machine Learning. 2001;45(1):5–32.

12. Kursa MB, Rudnicki WR. Feature selection with the Boruta package. J Stat Softw. 2010;36(11):1–13.
