# Supplementary figures and images for: Altered gut microbiota in female mice with persistent low body weights following removal of post-weaning chronic dietary restriction
Source: Genome Med. 2016 Oct 3;8:103. doi: 10.1186/s13073-016-0357-1 (PMC5048651; doi:10.1186/s13073-016-0357-1)

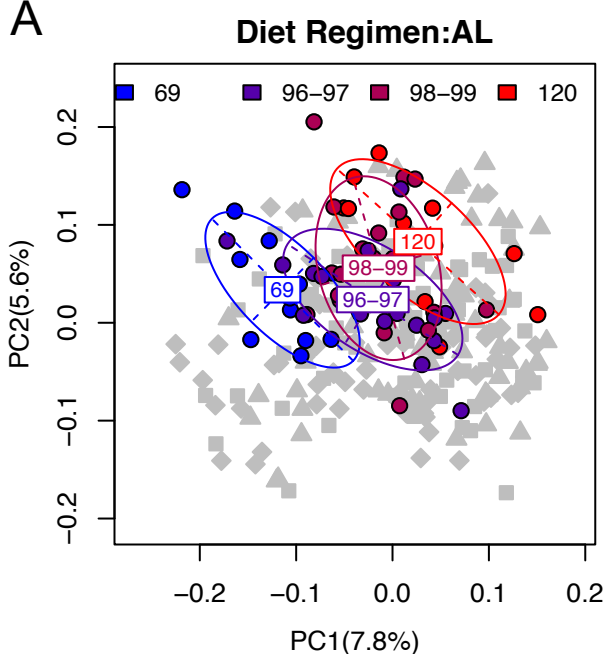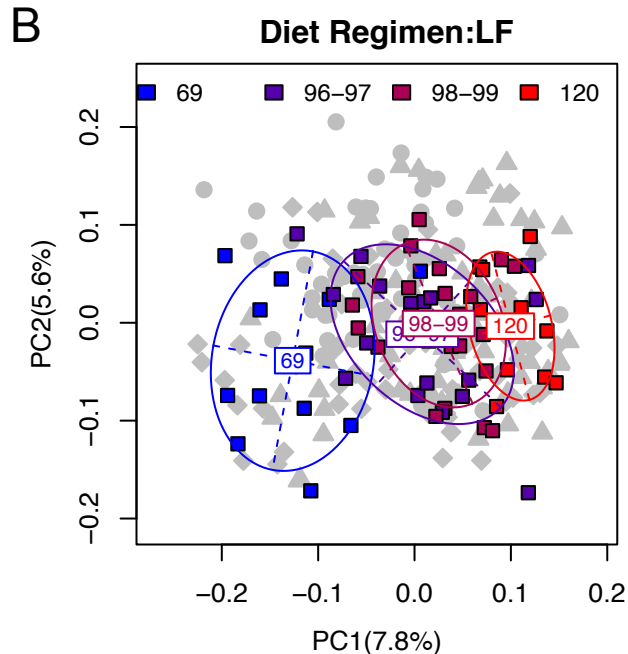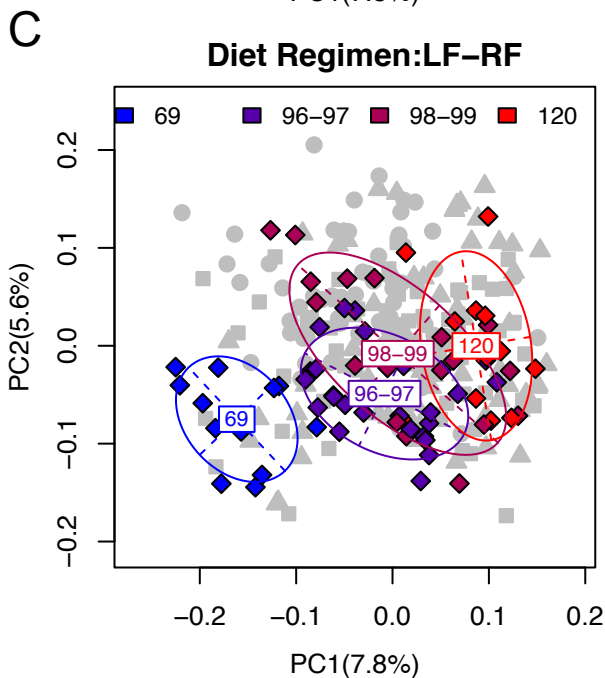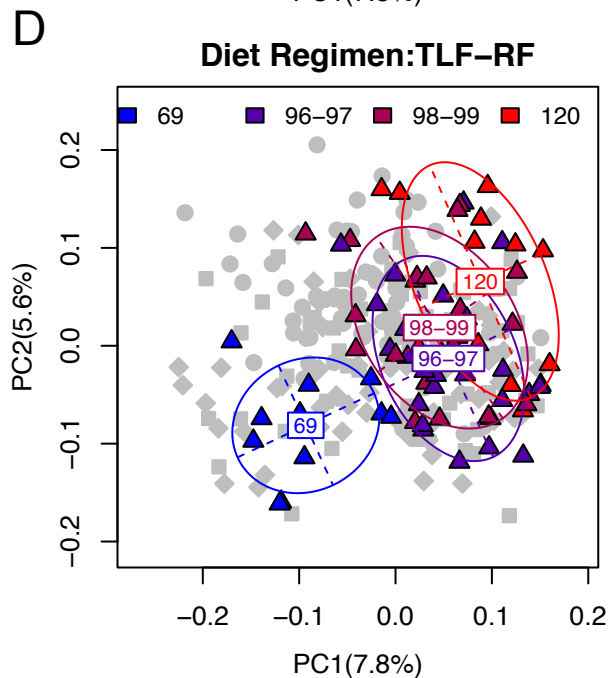

Supplement: Additional file 2: — The age effect on gut microbiota is consistent across diet groups. The first two PCs from the PCA on the unweighted UniFrac distance matrix are plotted. A–D Samples from different diet groups. Samples are color-coded by sampling days. The main axes of the ellipses correspond to the principal components of the group with the heights and widths representing variances in the corresponding components. (PDF 536 kb) [file 13073_2016_357_MOESM2_ESM.pdf]

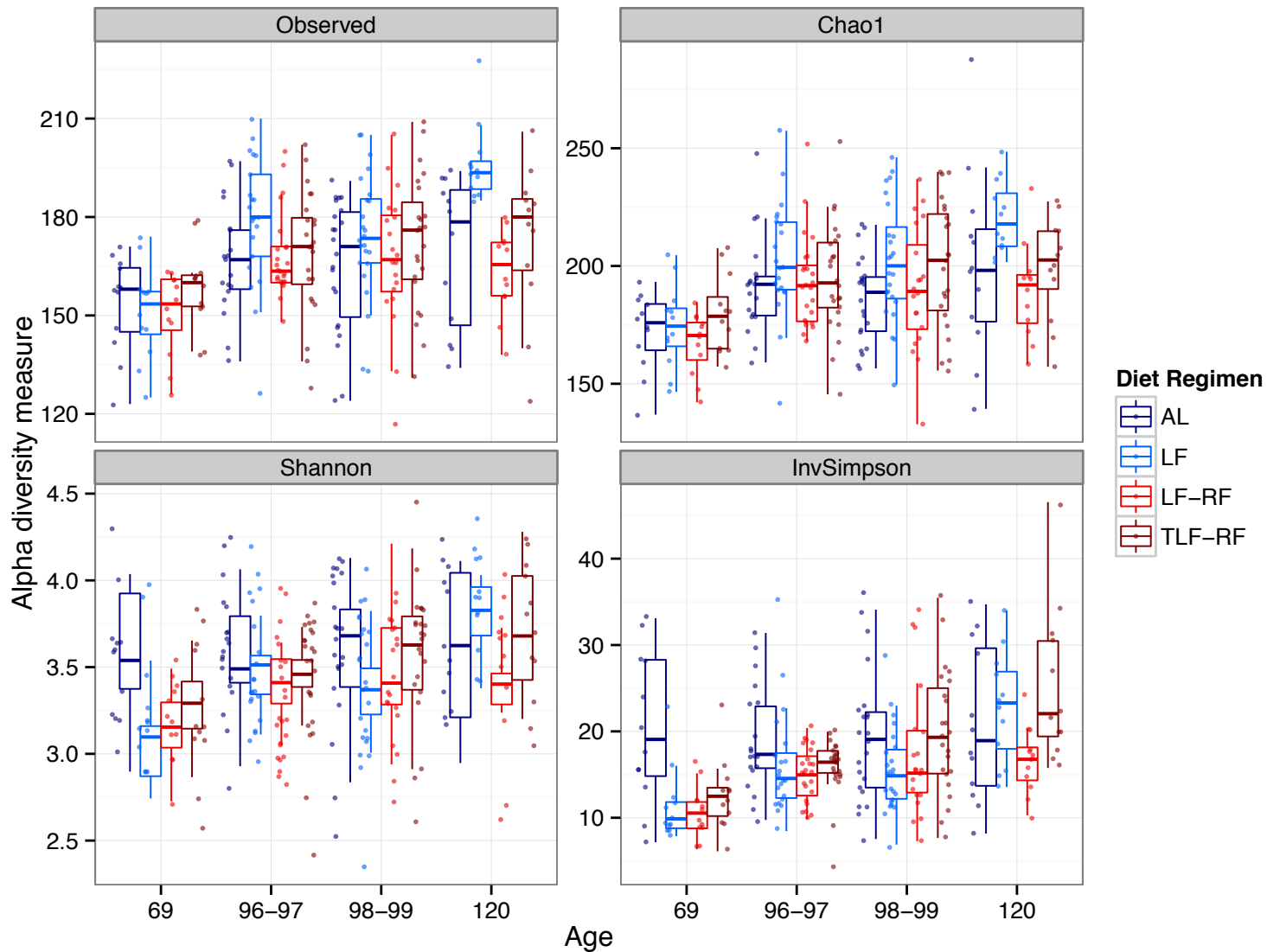

Supplement: Additional file 3: — Alpha diversity of the gut microbiota increases with age. OTU data were rarefied before calculating the diversity measures. Four diversity measures are shown: observed number of OTUs (Observed), Chao1 species richness estimator (Chao1), Shannon diversity index (Shannon), and inverse Simpson diversity index (InvSimpson). All diversity measures increased in LF, LF-RF, and TLF-RF mice, whereas only species richness indices (Observed and Chao1) increased in AL mice. (PDF 787 kb) [file 13073_2016_357_MOESM3_ESM.pdf]

A

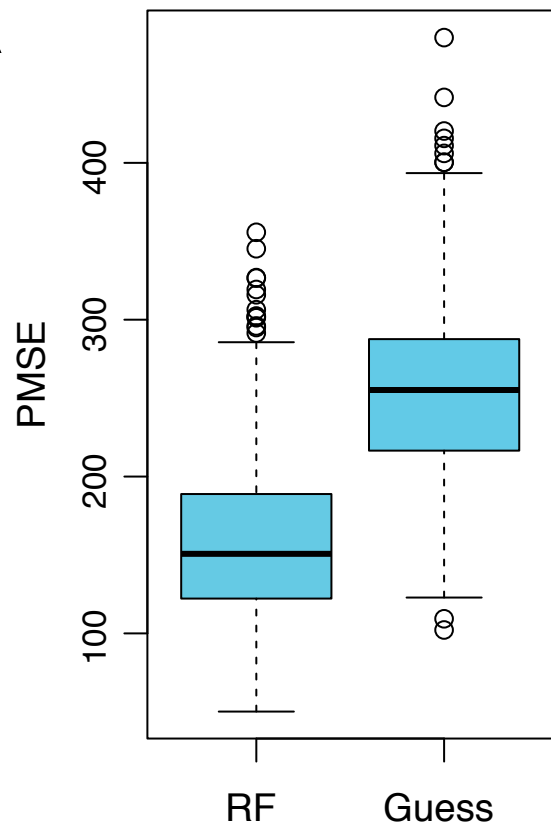

B

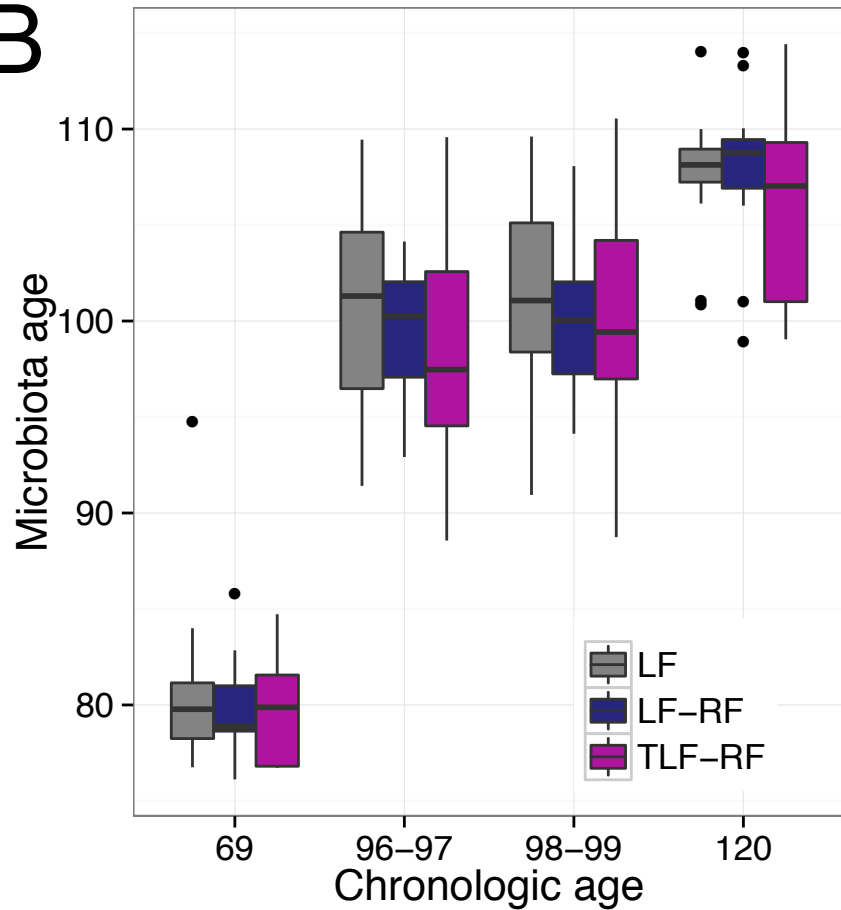

Supplement: Additional file 4: — Gut microbiota have strong age-predictive power. A Random Forests analysis achieves much smaller PMSE than the prediction based on the mean age in the training set (Guess). B The age of the microbiota samples was predicted using samples from AL diet group as the training set. The y axis represents the predicted age (microbiota age) by Random Forests analysis. Colors represent individual diet groups. (PDF 136 kb) [file 13073_2016_357_MOESM4_ESM.pdf]

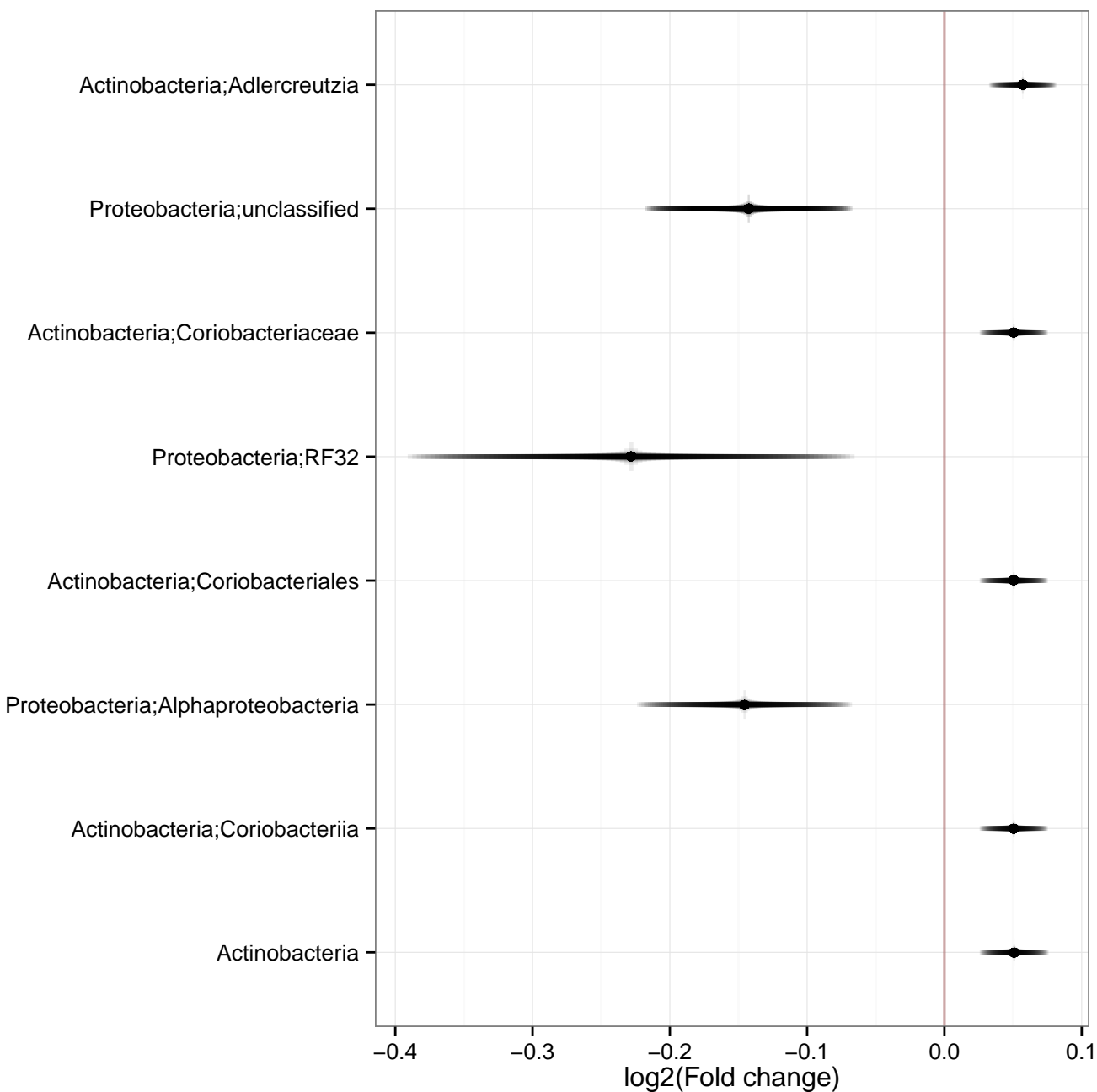

Supplement: Additional file 5: — Log2 fold change of the abundance of body weight-associated taxa in response to one unit change of body weights. (PDF 9 kb) [file 13073_2016_357_MOESM5_ESM.pdf]
